# Up-regulation of BMP-2 antagonizes TGF-β1/ROCK-enhanced cardiac fibrotic signalling through activation of Smurf1/Smad6 complex

**DOI:** 10.1111/j.1582-4934.2012.01538.x

**Published:** 2012-09-26

**Authors:** Shijun Wang, Aijun Sun, Lei Li, Gang Zhao, Jianguo Jia, Keqiang Wang, Junbo Ge, Yunzeng Zou

**Affiliations:** Shanghai Institute of Cardiovascular Diseases Zhongshan Hospital and Institutes of Biomedical Sciences Fudan UniversityShanghai, China

**Keywords:** BMP-2, TGF-β1, Smad6, Rho-associated kinase, cardiac fibrosis

## Abstract

Rho-associated kinase (ROCK) plays a critical role in pressure overload-induced left ventricular remodelling. However, the underlying mechanism remains unclear. Here, we reported that TGF-β1-induced ROCK elevation suppressed BMP-2 level and strengthened fibrotic response. Exogenous BMP-2 supply effectively attenuated TGF-β1 signalling pathway through Smad6-Smurf-1 complex activation. *In vitro* cultured cardiomyocytes, mechanical stretch up-regulated cardiac TGF-β1, TGF-β1-dependent ROCK and down-regulated BMP-2, but BMP-2 level could be reversed through blocking TGF-β1 receptor by SB-431542 or inhibition of ROCK by Y-27632. TGF-β1 could also activate ROCK and suppress endogenous BMP-2 level in a dose-dependent manner. Knock-down BMP-2 enhanced TGF-β1-mediated PKC-δ and Smad3 signalling cascades. In contrast, treatment with Y-27632 or SB-431542, respectively suppressed ROCK-dependent PKC-δ and Smad3 activation, but BMP-2 was only up-regulated by Y-27632. In addition, BMP-2 silencing abolished the effect of Y-27632, but not SB-431542 on suppression of TGF-β1 pathway. Further experiments showed that Smad6 Smurf1 interaction were required for BMP-2-evoked antagonizing effects. Smad6 overexpression attenuated TGF-β1-induced activation of PKC-δ and Smad3, promoted TGF-β RI degradation in BMP-2 knock-down cardiomyocytes, and could be abolished after knocking-down Smurf-1, in which Smad6/Smurf1 complex formation was critically involved. *In vivo* data showed that pressure overload-induced collagen deposition was attenuated, cardiac function was improved and TGF-β1-dependent activation of PKC-δ and Smad3 was reduced after 2 weeks treatment with rhBMP-2(0.5 mg/kg) or Y-27632 (10 mg/kg) in mice that underwent surgical transverse aortic constriction. In conclusion, we propose that BMP-2, as a novel fibrosis antagonizing cytokine, may have potential beneficial effect in attenuating pressure overload-induced cardiac fibrosis.

## Introduction

Cardiac and vascular fibrosis, mediated by pressure and volume overload, is one of the important causes responsible for the development of heart failure. Growing evidence indicates that pressure overload-mediated up-regulation of fibrosis-related cytokines, such as basic fibroblast growth factor (bFGF), connective tissue growth factor (CTGF) and transforming growth factor-β (TGF-β), could promote cardiac and vascular fibrosis [1, 2]. Pronounced cardiac hypertrophy and fibrosis were accompanied by significantly up-regulated expression of TGF-β and bFGF in 12-month-old spontaneously hypertensive rats (SHR) [3]. TGF-β1 overexpression in normal hearts induced atrial fibrosis and enhanced incidence of atrial fibrillation in mice [4], whereas blocking TGF-β1-dependent signalling improved cardiac function and inhibited cardiac fibrosis *in vivo* [5, 6]. In most cells, TGF-β1 binds to the ubiquitously expressed activin receptor like kinase-5 (ALK-5) receptor, and induces Smad3 activation and nucleus translocation [7, 8]. Activated Smad3 could promote multiple collagen proteins production and secretion, which results in subsequent myocardial and cardiovascular proliferation, migration and extra cellular matrix (ECM) deposition, finally leads to cardiac remodelling and heart failure. Therefore, modulating TGF-β or TGF-β-mediated signalling might be suitable strategies for preventing cardiac fibrosis.

Recent reports showed that bone morphogenetic proteins (BMPs), initially characterized as multi-potential family proteins regulating cell growth and differentiation, could reverse fibrosis progress [9, 10]. Overexpression BMP-2 in renal interstitial fibroblast cells effectively antagonized TGF-β1-mediated fibrosis by enhancing the catabolism of type I TGF-β receptors (TGF-β RI). Lung epithelial cells expressing high levels of Gremlin, a BMP4 inhibitor, were more susceptible to epithelial-to-mesenchymal transition (EMT) after exposure to TGF-β1, Gremlin overexpression significantly enhanced the fibrotic response *via* BMP-4 signalling [11]. Other studies also demonstrated that BMP-7 could reverse chronic renal injury through counteracting TGF-β1 induced EMT and TGF-β1 dependent collagen protein secretion [10, 12]. Therefore, we tested the hypothesis that BMP-2 could attenuate pressure overload-induced cardiomyocytes fibrosis by antagonizing TGF-β1-mediated fibrotic signalling. The present study indicated that pressure overload activated ROCK and suppressed the endogenous BMP-2 expression, the fibrotic response activated by PKC-δ and Smad3 could be effectively suppressed by exogenous BMP-2 supplement. BMP-2-mediated antifibrotic effect was linked with Smad6 activation, and Smad6 activation reduced the phosphorylation levels of p-PKC-δ and p-Smad3. *In vivo* studies also suggested that pressure overload-induced cardiac fibrosis could be attenuated by inhibiting ROCK activity or increasing BMP-2 level.

## Materials and methods

### Reagents and plasmids

Recombinant human BMP-2 (Cas # 120-02) was purchased from Pepro Tech Inc (Rocky Hill, NJ, USA), SB431542 (Cas# 301836-41-9), GF109203X (Cas# 133052-90-1) from Sigma Aldrich (St. Louis, MO, USA), Y-27632 (Cas # 146986-50-7) from Merck (Whitehouse Station, NJ, USA), anti-ROCK1 (#4035, Cell Signaling Technology, Beverly, MA, USA), anti-p-PKC-δ^Tyr 155^, anti-p-Smad3^Ser 423/425^, anti-Smad6 (sc-13048), anti-Smad7 (sc-11392) and anti-TGF-β RI (sc-398) from Santa Cruz Biotechnology (Santa Cruz, CA, USA), anti-Smurf-1(ab57573) from Abcam plc (Cambridge, UK). The Smad6 expression plasmid was constructed by subcloning the open reading frame (ORF) and promoter sequences into the same expression pEZ-M29 vector (Gene Copoeia™, Guangzhou, China).

### Animal models

Male WT aged 10 weeks, C57/BL6 mice (purchased from Animal Administration Center, Shanghai, China) were used in the present study. BMP-2 (0.5 mg/kg body weight) was implanted subcutaneously into the mice using Alzet osmotic mini pumps (DURECT, Cupertino, California). Pressure overload was produced by constriction of transverse aorta (TAC) for 2 weeks as described previously. All of the animal experiments were performed in compliance with the Guide for the Care and Use of Laboratory Animals published by the US National Institutes of Health (NIH Publication No.85-23, revised 1996) and was approved by the guidelines of Fudan University.

### Cultures and mechanical stretch of cardiomyocytes

Neonatal ventricular cardiomyocytes were cultivated as we previously described [13]. Cardiomyocyte purity was identified by immunofluorescence after staining with mono-clonal antibodies specific for cardiac α-myosin heavy chain (α-MHC). Cardiomyocytes were cultivated in serum and antibiotic-free condition before mechanical stretch, SB431542 (10 μM), Y-27632 (10 μM) and BMP-2 (50 ng/ml) were pre-administered into the medium. After stretching for 12, 24 and 48 hrs, the cells were collected for the extraction of protein and total RNA for the following experiments:

### RNA extraction and qRT-PCR

Total RNA was isolated from cardiomyocytes using TRIZOL reagent (Invitrogen) according to the manufacturer's instructions. Then cDNA was obtained from total RNA by reverse transcription with SUPERSCRIPTTM III First-Strand Synthesis System (Invitrogen, Carlsbad, CA, USA). Quantitative Real-time PCR was performed on Rotor-Gene 3000(Corbett Research, Australia) using the standard curve method and normalized by the housekeeping gene GAPDH. PCR condition was 35 cycles at 94°C for 30 sec., 54°C for 30 sec., 72°C for 45 sec. The primers used were as follows: BMP-2, 5′-TTTCCTGTGACCAGACTATTG-3′, 5′-GTTTGGCTTGACGCTTTT-3′; TGF-β1, 5′-AATGGTGGACCGCAACAAC-3′, 5′-TGAGCACTGAAGCGAAAGC-3′; CTGF, 5′-CTTCGGTGGGTCCGTGTA-3′, 5′-GCAGTTGGCTCGCATCAT-3′; GAPDH, 5′-GCCTTCCGTGTTCCTACC-3′, 5′-GCCCCTCCTGTTGTTATG-3′.

### Co-immunoprecipitated (IP) analysis

Serum-free cultured cardiomyocytes were stimulated by rhBMP-2 (100 ng/ml) for various check time points, then cells were lysed with extraction buffer (20 mMHEPES (pH7.5), 150 mM NaCl, 12.5 mM β-glycerophosphate, 1% Triton X-100, 1.5 mM MgCl2, 2 mM EGTA, 10 mM NaF, 2 mM DTT, 1 m Na_3_VO_4_ and 1 m phenylmethylsulfonyl fluoride). Endogenous Smurf-1 was precipitated using mouse monoclonal antibody against Smurf-1, immuno-complexes were collected on protein A-Sepharose beads and washed three times with washing buffer (20 mM HEPES (pH7.5), 500 mM NaCl, 10 mM MgCl2). The beads were eluted with SDS/PAGE loading buffer after extensive washes with Tris-buffered saline and were subjected to SDS/PAGE and Western blotting.

### Western blot analyses

Equal amount of cardiomyocyte lysates were separated on 12% PAGE and transferred into PVDF membranes (Millipore Corp., Bedford, MA, USA). Then, the membranes with blotted protein were blocked for 1 hr in 0.1% TBS-Tween 20 (TBS-T), containing 5% bovine serum albumin, followed by probing with anti-ROCK1 (1:1000), anti-p-PKC-δ Tyr^155^ (1:200), anti-p-Smad3 Ser^423/425^ (1:200), anti-Smad6 (1:200), anti-Smad7 (1:200) and anti-TGF-β RI (1:1000) antibodies at 4°C overnight. The immunoreactive proteins were visualized by the luminescence method (Super Signal West Pico Chemiluminescence Substrate kit, Thermo Scientific Pierce, Waltham, MA, USA) with HRP-conjugated anti-IgG (1:5000 dilution), and quantified by the ChemiDoc™ system (Bio-Rad, Hercules, CA, USA). β-actin was used as the loading control for each lane.

### RNA interference

The short interfering RNA (siRNA) against BMP-2 (NM_017178, Genebank) was designed and synthesized by Shanghai Gene Chem. Inc. (Shanghai, China). The siRNA sequence was as follows: 5′-ccgg-aaCCATGCCATAGTGCAGACTTTCAAGAGA.

-(loop)-AGTCTGCACTATGGCATGGtt-TTTTTg-3′. The siRNA against Smad3, Smad6, Smurf-1 as well as scrambled non-targeting siRNA were purchased from Santa Cruz Biotechnology. SiRNA transfection was performed with transfection medium according to the manufacturer's protocol. 24 hr after transfection, cell lysates were subjected to RT-PCR and Western blot analysis for identifying gene expression.

### Statistical analysis

Data were presented as mean ± S.E.M. for at least four repeated individual experiments for each group. Statistical analyses were performed using SPSS11.0 software. Correlations between sample groups and molecular parameters were calculated by one-way anova for independent samples. A *P* value < 0.05 was considered statistically significant.

## Results

### Cardiac BMP-2 transcriptional level was inhibited by mechanical stretch-induced ROCK up-regulation

**Fig 1:**
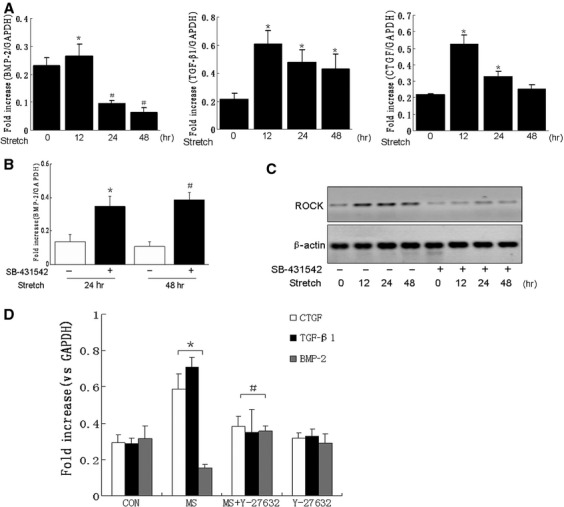
Mechanical stretch-induced BMP-2 inhibition through TGF-β1-dependent ROCK signalling. (A) mRNA levels of BMP-2, TGF-β1, CTGF were detected by quantitative PCR after stretching cardiomyocytes for 0, 12, 24, 48 hrs, respectively. (B) quantitative PCR analyses of cardiac BMP-2 mRNA level after stretching for 24 and 48 hrs in the absence or presence of SB431542 (10 μM). (C) Western blotting analyses of stretch-induced ROCK expression in the absence or presence of SB431542 (10 μM) for the indicated time points. (D) Mechanical stretch-induced mRNA levels of BMP-2, TGF-β1 and CTGF in the absence or presence of Y-27632 (10 μM). Bar graphs represented the means ± S.E.M of data from independent sample/group (*n* = 4). *P < 0.05, *versus* Control group; #*P* < 0.05, *versus* MS group. (MS: mechanical stretch).

BMP-2 was functionally expressed in neonatal ventricular cardiomyocytes which was required for early heart development [14, 15]. To understand the functional role of BMP-2 in pressure overload-induced cardiac fibrosis, we tested a number of TGF-β family cytokines including BMP-2 in cultured cardiomyocytes that underwent mechanical stretch. The results showed that expression of TGF-β1 and CTGF (two of the pro-fibrotic factors) was increased after stretching for 12 hr, and TGF-β1 level was maintained at 24 and 48 hr; but endogenous BMP2 mRNA level was significantly reduced after stretching for 24 hr (Fig. 1A). To explore whether enhanced TGF-β1 signal caused the inhibition of BMP2, we examined stretch-induced-BMP2 expression after blocking TGF-β1 signalling by SB431542 (10 μM), the specific inhibitor of TGF-β type I receptor (TGF-β RI). As shown in Figure 1B, reduced BMP2 was up-regulated again after blocking TGF-β RI at 24 and 48 hr, respectively. In addition, TGF-β1-dependent ROCK was induced by mechanical stretch and suppressed by pre-treating with SB431542 in a time-dependent manner (Fig. 1C). To further confirm whether TGF-β1-dependent up-regulation of ROCK contributed to BMP2 inhibition, we inhibited ROCK activity in cardiomyocytes using Y-27632 (10 μM), a Rho-kinase inhibitor. The enhanced transcriptional level of TGF-β1 and CTGF induced by mechanical stretch was down-regulated after treating with Y-27632, and BMP-2 was up-regulated by Y-27632 (Fig. 1D). These data indicated a critical role for ROCK in suppressing BMP-2 expression, which resulted in a positive feedback loop of mechanical stretch-induced TGF-β1 signalling.

### BMP-2 knock-down enhanced TGF-β1-mediated PKC-δ and Smad3 signalling cascade

**Fig 2:**
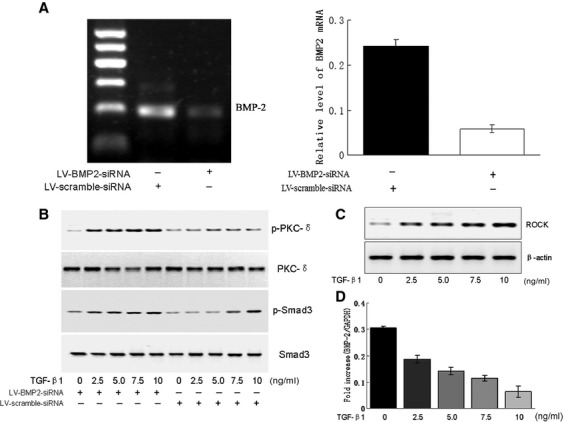
Knocking down BMP-2 enhanced TGF-β1-mediated phosphorylation levels of PKC-δ and Smad3. (A) Quantitative PCR analyses of BMP-2 level in cardiomyocytes treated with special lentivirus-mediated anti-BMP-2 siRNA (LV-BMP2-siRNA) or LV-scramble siRNA, respectively for 24 hr. (B) TGF-β1 (0∼10 ng/ml)-induced phosphorylation levels of PKC-δ at Tyr^155^ and Smad3 at Ser ^423/425^ were determined by Western blotting in cultured cardiomyocytes pre-treated with LV-BMP2-siRNA or LV-scramble-siRNA for 24 hr. (C) Western blotting analyses of the protein level of ROCK induced by TGF-β1 (0∼10 ng/ml) for 24 hr. (D) Quantitative PCR analyses of the mRNA level of BMP-2. Each bottom blot indicated equal loading of proteins normalized by total PKC, Smad3 or β-actin. Data were representative as means ± S.E.M. of *n* = 4 independent experiments.

To prove whether BMP-2 inhibition promoted TGF-β1-mediated fibrotic signalling pathway, we examined TGF-β1-induced activation of PKC-δ and Smad3, two important signal molecules that induced myocardial fibrosis [16, 17], after knocking down BMP-2 in cardiomyocytes. The transfection efficiency of the lentivirus-mediated anti-BMP-2 RNA interference (RNAi) was confirmed and evaluated by quantitative PCR analyses (Fig. 2A) after transfection for 72 hr in cultured cardiomyocytes. Compared with that induced by lentivirus-scramble siRNA, the phosphorylation levels of PKC-δ and Smad3 were up-regulated in lentivirus-BMP-2 siRNA-transfected cardiomyocytes (Fig. 2B). p-PKC-δ and p-Smad3 were also increased at high dose (10 ng/ml) of TGF-β1 pre-treated cells transfected with lentivirus-scramble siRNA (Fig. 2B, the last lane). Specially, the kinetic response for TGF-β1-invoked p-PKC-δ phosphorylation was examined in a time-dependent course, data showed that PKC-δ activation reached the peak value at about 15 min. after stimulation by TGF-β1 (Fig. S1). Notably, we also observed a dosage-dependent reduction in BMP-2 expression and increase in ROCK expression induced by TGF-β1 in cardiomyocytes transfected with lentivirus-scramble siRNA (Fig. 2C and D).

### ROCK inhibition abrogated PKC-δ and Smad3 activation evoked by TGF-β1

**Fig 3:**
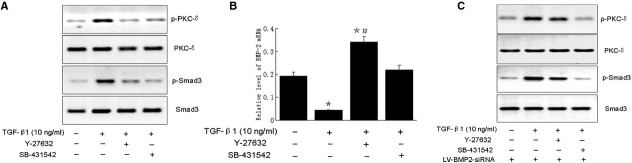
ROCK inhibition abrogated PKC-δ and Smad3 activation evoked by TGF-β1. (A) The effect of TGF-β1 (10 ng/ml) on the phosphorylation levels of PKC-δ and Smad3 in the presence or absence of Y-27632 or SB-431542, respectively for 24 hr, was determined by Western blotting, all the blots were normalized by total PKC-δ or Smad3 levels. (B) The effect of TGF-β1 (10 ng/ml) on the transcriptional levels of BMP-2 in the presence or absence of Y-27632 or SB-431542, respectively for 24 hr, was evaluated by quantitative PCR. (C) Western blotting analyses of TGF-β1 (10 ng/ml)-induced activation of PKC-δ and Smad3 with or without treatment of Y-27632 or SB-431542 for 24 hr in BMP-2 knock-down cardiomyocytes. Data were representative as means ± S.E.M. of *n* = 4 independent experiments. *P < 0.05, *versus* Control group; #*P* < 0.05, *versus* TGF-β1 (10 ng/ml) group.

Considering that BMP-2 might be inhibited by ROCK elevation (Fig. 2D), we further analysed the role of ROCK kinase in TGF-β1-mediated PKC-δ and Smad3 signal transduction. As shown in Figure 3A, TGF-β1(10 ng/ml) induced a robust increase in phosphorylation levels of PKC-δ and Smad3, which were markedly reduced after inhibition of ROCK with treatment of Y-27632(10 μM) or SB431542(10 μM), respectively. Simultaneously, a significant increase in BMP-2 expression was observed in cardiomyocytes stimulated by Y-27632, but not by SB431542 (Fig. 3B). Next, we inhibited endogenous BMP-2 by transfecting lentivirus-BMP-2 siRNA, and determined the effect of Y-27632 or SB431542 on TGF-β1-mediated fibrotic signalling. Activated PKC-δ and Smad3 were not affected by Y-27632, but suppressed by SB431542 in BMP-2 knocked-down cardiomyocytes (Fig. 3C), which indicated that TGF-β1 mediated PKC-δ and Smad3 signal transduction *via* TGF-β type I receptor; these signalling cascades could be regulated by BMP-2 dependent ROCK inhibition. Furthermore, we explored the effects of PKC-δ and Smad3 on intercellular ROCK levels. Results indicated that TGF-β1-mediated ROCK elevation was not affected by inhibiting PKC-δ with GF109203X, or knocking-down with Smad3 siRNA (Fig. S2).

### Smurf1/Smad6 interaction was required for BMP-2 dependent suppression of PKC-δ and Smad3 signalling cascade

**Fig 4:**
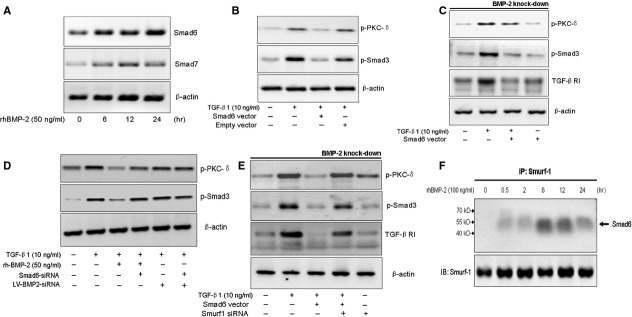
BMP-2 inhibited TGF-β1-mediated PKC-δ and Smad3 signalling cascade through Smurf1/Smad6 interaction and activating Smad6. (A) Effect of rhBMP-2 (50 ng/ml) on time-dependent Smad6 and Smad7 expression in cultured cardiomyocytes. (B) Effect of TGF-β1 (10 ng/ml) on phosphorylation levels of PKC-δ and Smad3 in cells after 24 hr transfecting with or without Smad6 expression plasmid or pEZ-M29 plasmid, respectively. (C) Effect of TGF-β1 (10 ng/ml) on expression of TGF-β RI receptor and phosphorylated PKC-δ and Smad3 in the presence or absence of Smad6 expression plasmid in BMP-2 knock-down cardiomyocytes. (D) The effects of rhBMP-2 (50 ng/ml) supply or BMP-2 knock-down on TGF-β1 (10 ng/ml)-induced activation of PKC-δ and Smad3 in cells after 24 hr transfecting with or without Smad6 siRNA. (E) Effect of TGF-β1 (10 ng/ml) on expression of TGF-β RI receptor and phosphorylated PKC-δ and Smad3 in BMP-2 knock-down cardiomyocytes in the absence or presence of Smurf1 siRNA. Each bottom blot indicated equal loading of proteins normalized by β-actin. (F) Expression of Smad6 was examined in immunoprecipitated (IP) complex with anti-Smurf-1 antibody, and Smurf-1 in total cell lysates.

ROCK-induced BMP-2 suppression critically involved in TGF-β1 mediated PKC-δ and Smad3 signal transduction, which raised another question as how did BMP-2 regulate TGF-β1-dependent fibrotic signalling. Previous studies showed that two inhibitory Smads (Smad6 and 7) played important roles in inhibiting TGF-β superfamily-induced signalling transduction [18, 19], and Smad6 was critically involved in the development and homeostasis of the cardiovascular system [20]. To analyse whether Smad6 or Smad7 was required for BMP-2-mediated inhibition of TGF-β1 signalling, we first examined the protein levels of Smad6 and Smad7 induced by rhBMP-2 (50 ng/ml). As demonstrated in Figure 4A, Smad6 expression was significantly increased when compared with Smad7 in response to rhBMP-2 for more than 6 hr. Next, we examined the effect of Smad6 on TGF-β1-dependent activation of PKC-δ and Smad3 in Smad6 overexpressed cardiomyocytes (Fig. S3).TGF-β1(10 ng/ml)-mediated phosphorylation levels of PKC-δ and Smad3 were suppressed by transient overexpression of Smad6 (Fig. 4B). Increasing Smad6 also attenuated TGF-β1-induced activation of p-PKC-δ and p-Smad3 in BMP-2 knock-down cardiomyocytes (Fig. 4C). In addition, Smad6 expression plasmid attenuated the receptor level of TGF-β RI (Fig. 4C). Moreover, we examined the role of TGF-β1 in PKC-δ and Smad3 signalling cascades after knocking-down Smad6 in either BMP-2 treated or BMP-2 knock-down cardiomyocytes. As shown in Figure 4D, BMP-2-induced inhibitory effect on TGF-β1-dependent activation of p-PKC-δ and p-Smad3 could be reversed by silencing Smad6, but silencing Smad6 did not affect the phosphorylation levels of PKC-δ and Smad3 in BMP-2 knock-down cardiomyocytes.

Smad ubiquitin regulatory factors (Smurf1), members of the Hect-domain family of E3 ubiquitin ligases, were previously reported as an important regulator for receptor degradation of TGF-β RI. To identify whether Smad6-mediated TGF-β RI degradation was in a ubiquitin proteasome-dependent manner, the effect of Smad6 on TGF-β1 signal was further examined in cells knock-down Smurf1 by special siRNA (Fig. S4). Results showed that overexpressing Smad6 failed in inhibiting receptor degradation of TGF-β RI and TGF-β1-induced up-regulation of p-PKC-δ and p-Smad3 (Fig. 4E) after silencing Smurf1. Notably, immunoprecipitation analysis showed that the amount of Smad6 coprecipitated with Smurf1 was significantly increased at 6 hr after stimulating with rhBMP-2 (100 ng/ml) in the anti-Smurf1 immunoprecipitates (Fig. 4F). These data strongly suggested that Smad6/Smurf1 complex formation as critical signal transducer linking BMP-2-induced inhibitory effect on TGF-β signal. BMP-2 regulated TGF-β1-mediated signalling pathway possibly through activating Smad6/Smurf1-mediated ubiquitination system and degradation of TGF-β RI.

### Up-regulation of BMP-2 level attenuated pressure overload-induced cardiac fibrosis *in vivo*

**Fig 5:**
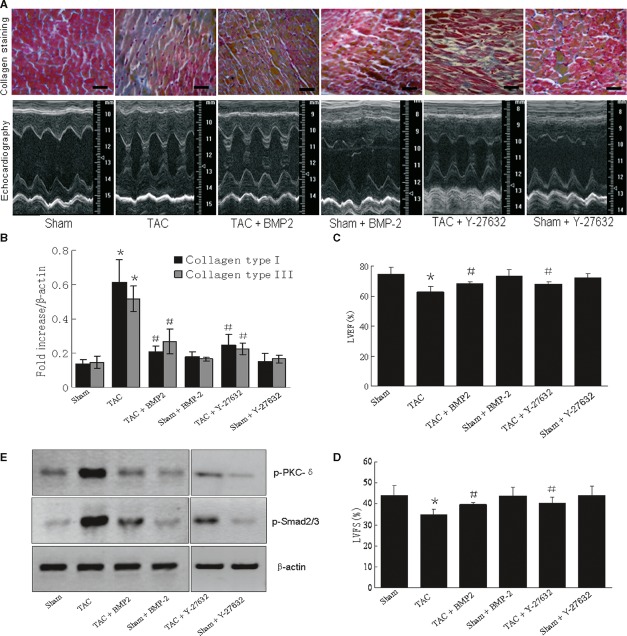
Effect of BMP-2 and Y-27632 on TAC-evoked fibrotic response and cardiac function *in vivo*. (A) Representative images of collagen staining (scale bar: 30 μM) and echocardiographic M-mode tracings were evaluated at 2 weeks after TAC or sham operation of mice with or without treatment of rhBMP-2 (0.5 mg/kg body weight) or Y-27632 (10 mg/kg body weight). (B) Quantitative analyses of the transcriptional levels of collagen type I and type III in different treated groups. (C–D) Echocardiographic parameter analyses of left ventricular eject fraction (LVEF%) and left ventricular fractional shortening (LVFS%) in mice with different treatment. (E) Phosphorylation levels of PKC-δ and Smad3 in mice from different groups. Bar graphs represented as means S.E.M. of data from independent mouse/group (*n* = 6). *P < 0.05, *versus* Sham group; #*P* < 0.05, *versus* TAC group.

We next investigated the physiological significance of BMP-2 in pressure overload-induced cardiac fibrosis *in vivo*. Male wide-type C57/BL6J mice were treated with BMP-2 (0.5 mg/kg body weight) or Y-27632 (10 mg/kg body weight) by implanting a mini osmotic pump 24 hr after surgical transverse aortic constriction (TAC). As shown in Figure 5A, inhibition ROCK activity with Y-27632 effectively suppressed pressure overload-induced collagen deposition in TAC mice at 2 weeks after TAC; however, exogenous supply of BMP-2 could also reduce the formation of collagens as well as decreasing collagen type I and type III gene expression (Fig. 5A and B). In addition, echocardiographic measurement showed that both the left ventricular end systolic posterior wall thickness (LVPWs) and left ventricular end-diastolic posterior wall thickness (LVPWd) increased by 2 weeks of TAC, was attenuated by BMP-2 or Y-27632 treatment, respectively (Fig. 5A bottom line). Impaired cardiac function in TAC mice at 2 weeks after TAC was significantly improved by BMP-2 or Y-27632 treatment. The left ventricular ejection fraction (LVEF%) increased (≍9%, 68.24% ± 1.17%, *P* < 0.05) by BMP-2 and (≍8%, 67.67% ± 3.43%, *P* < 0.05) by Y-27632; the left ventricular fractional shortening (LVFS%) improved by (39.76% ± 0.97%, *P* < 0.05) and (39.91% ± 1.04%, *P* < 0.05) in TAC mice pre-treated with BMP-2 or Y-27632, respectively (Fig. 5C and D). Westernblot analyses showed that TAC-induced up-regulation of the phosphorylation levels of PKC-δ and Smad3 were markedly reduced by BMP-2 or Y-27632 pre-treatment in hearts underwent TAC for 2 weeks (Fig. 5E). Collectively, these results strongly suggested that pressure overload-induced cardiac fibrosis was triggered by TGF-β1 mediated p-PKC-δ and p-Smad3 signalling cascade, which could be attenuated by enhancement of BMP-2 level *in vivo*, possibly *via* antagonizing ROCK-dependent TGF-β1 signalling pathway.

## Discussion

Cardiac fibrosis, impaired regulation of the wound healing response during heart remodelling, is regarded as one of the major causes of heart failure. In the previous studies, pressure overload-induced TGF-β1 signalling has been implicated in the primary pathogenesis of fibrotic disorders [7, 21]. Transfection *in vitro* and administration *in vivo* of recombinant BMPs molecules significantly inhibited EMT and the progression of cardiac fibrosis in pressure overload models [10]; however, the potential mechanism is poorly understood. In this study, we explored the potential mechanisms for BMP-2-induced inhibitory effect on TGF-β1-mediated cardiac fibrotic response in cardiomyocytes, in which Smurf1-dependent Smad6 activation is critically involved.

BMP-2 is previously reported to be effective in preventing renal fibrosis [9], the underlying mechanism is linked with the reduction of TGF-β RI and down-regulation of Smads signalling. Despite the fact that BMP-2 belongs to TGF-β superfamily member cytokine, BMP-2 and TGF-β1 exert different roles in cardiac formation. BMP-2 contributes primarily to terminal differentiation of cardiomyocytes, not to the expression of transcriptional regulators for cardiac fates [15, 22]. BMPs and TGF-βs family cytokines interact with each other through regulating their unique receptors. BMP7 affects two distinct TGF-β type I receptors, ALK-5 and ALK-1, which are required for TGF-β1-mediated Smad3 phosphorylation and nuclear translocation, inhibition of TGF-β receptor greatly attenuates Smad3-dependent cardiac fibrosis [21, 23, 24]. In the present study, we revealed that TGF-β1-induced ROCK elevation suppressed the BMP-2 expression, constantly enhanced ROCK activity under pressure overload possibly blocked BMP-2 signal and strengthened TGF-β biased fibrotic signalling. Previous studies also demonstrated that inhibition of ROCK by Statins could enhance cellular BMP-2 mRNA level and promote osteoblast differentiation by rescuing BMP-2-induced Runt-related gene2 (Runx2) and ALP activity that controlled by Ras/Rho/MAPK pathway [25, 26]. Y-27632, another Rho kinase inhibitor, stimulates bone formation *via* activating the BMP-4 signal [27]. In our study, the observed effects of ROCK inhibitor, Y-27632, were achieved through abolishing the suppression effect of ROCK on endogenous BMP-2. TGF-β1 signal activated both Smad3/PKC-δ and ROCK cascades, the latter response could inhibit endogenous BMP-2 elevation. Suppressed endogenous BMP2 levels induced by ROCK activation could, to some extent, be improved by exogenous BMP2 supply *via* activating Smurf1/Smad6 downstream signalling pathway, which antagonized TGF-β1-mediated Smad3/PKC-δ signal cascades.

In addition, our present data showed that SB431542, the specific inhibitor of ALKs, could significantly inhibit increased ROCK expression and reverse attenuation of BMP-2 level, suggesting that TGF-β1 might be the first candidate for stretch-induced ROCK activation, and BMP-2 level could be regulated by enhanced ROCK, but we could not exclude the possible roles for other cytokines or molecules, which might bind to TGF-β type I receptor, activate ROCK and suppress BMP-2.

It is known that pressure overload could enhance the TGF-β1 signalling, including phosphorylation of the two major cellular pro-fibrotic effectors, PKC-δ and Smad3, increasing the deposition of ECM, collagens and fibronectin in cardiomyocytes [16, 17]. Multiple lines of evidence suggest that BMPs could oppose fibrogenic activity through these cascades. In our study, we showed that BMP-2 could effectively inhibit the activation of PKC-δ and Smad3 induced by TGF-β1, and we further demonstrated that BMP-2 inhibited the TGF-β1-dependent signal transduction *via* Smurf-1-dependent Smad6 activation. In previous studies, BMP-2 was shown to reverse TGF-β1-mediated increasing level of p-Smad3 and nuclear translocation of Smad4 in renal fibroblasts [9]. One minor limitation of this study is that Smad3 knockout mice are not available in our laboratory till now, the related investigation about BMP-2 effect on Smad3^−/−^ is not involved. Furthermore, BMP-7 is reported to attenuate Smad3-mediated type I collagen and fibronectin through inhibitors of differentiation 2 (Id2) in myofibroblasts [12]. Of note, heterodimers between BMP-2 and BMP-7 behaved with higher combined affinity for both type I and type II receptors and thus facilitated the formation of active signalling complexes [28, 29]. Further investigation is warranted to see if BMP-7 is required for coordinately regulating BMP-2-mediated TGF-β1 signalling.

In the current study, we also showed that Smurf1 was critically involved in BMP-2-induced degradation of TGF-β RI. Previous study demonstrated that Smurf-1-dependent Runx2 degradation was required for dynamic equilibrium of BMP-2 induced Smads signalling [30]. In addition, Smad6 and Smad7, two important I-Smads activated by Smurf1, might play critical roles in BMP-2 signal *via* mediating ubiquitination of target protein [31, 32]. Here, we observed that BMP-2 could up-regulate the expression of Smad6 but not Smad7 in cultured cardiomyocytes. Increased Smad6 could then be accumulated and activated by Smurf-1, which is required for Smad6-dependent TGF-β RI degradation and inhibition of TGF-β1-mediated PKC-δ and Smad3 signalling cascades. Importantly, Smad6 is required for regulation of BMP-2 mediated vascular function through accelerating Tbx6 and Runx2 ubiquitination [30, 33]. Smad6 knockout mice developed multiple cardiovascular abnormalities [20]. Smad6 may regulate and balance the intracellular Smads signalling cascades *via* recruitment of multiple Smurf proteins and activation of the E3 ubiquitin-protein ligase system. Dysfunction of proteasome degradation system in the absence of Smad6 might cause abnormality for the development and homeostasis of the cardiovascular system. In this study, we showed that Smurf1-Smad6 interaction was required for Smad6 activating to exert the effect on BMP-2 signal. Knock-down Smurf1 in Smad6 expressed cardiomyocytes abolished BMP-2-induced antagonizing effect on targeting TGF-β RI degradation and TGF-β1-dependent PKC-δ and Smad3 fibrotic signalling pathway.

**Fig 6:**
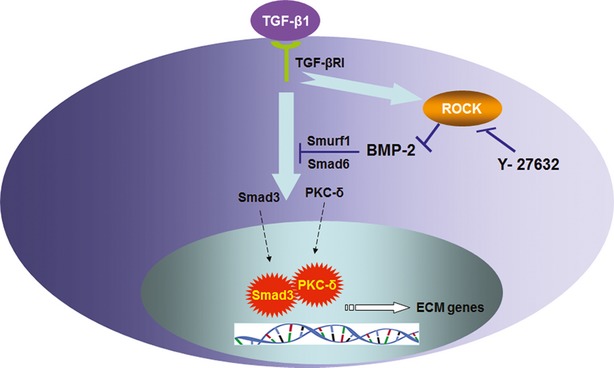
Schematic representation of a unified view of mechanism for BMP-2 mediated protective effect against TGF-β1-induced cardiac fibrotic response.

In conclusion, our findings provided novel insight into the potential mechanisms for BMP-2 mediated antagonizing effect on pressure overload-induced cardiac fibrosis. We have demonstrated that TGF-β1-induced elevation of ROCK level suppresses BMP-2 signal, and promotes fibrosis through phosphorylation of PKC-δ and Smad3; such effect could be reversed by increasing BMP-2 level *in vitro* and *in vivo*. Furthermore, Smurf1/Smad6 signal complex formation contributes to BMP-2-mediated inhibitory effect on TGF-β1 signal; Smurf1-dependent Smad6 activation attenuates TGF-β1-induced expression of phosphorylated PKC-δ and Smad3, and collagen deposition (Fig. 6). Our results might provide a new theoretical basis for developing therapeutic agents against cardiac fibrosis.

## Supporting information

**Figure S1** TGF-β1(2.5 ng/ml)-induced kinetic response of PKC activation was represented by time course of phosphorylation levels of PKC-δ after normalized by total PKC-δ.

**Figure S2** Western blotting analyses of TGF-β1(10 ng/ml)-mediated ROCK expression in the absence or presence of GF109203X (1 μM) or Smad3 siRNA.

**Figure S3** Immunoblots presented Smad6 expression from cardiomyocytes transfected with Smad6 plasmid or empty plasmid.

**Figure S4** Immunoblots presented Smurf-1 expression from cardiomyocytes transfected with Smurf-1 siRNA or scramble siRNA.

**Figure S5** TGF-β1(10 ng/ml)-evoked ROCK expression and phosphorylation levels of LIMK (phosphorylated by Thr505 and Thr508 residues) in the absence or presence of Y-27632 (10 μM).
