# Supplementary figures and images for: Up-regulation of BMP-2 antagonizes TGF-β1/ROCK-enhanced cardiac fibrotic signalling through activation of Smurf1/Smad6 complex
Source: J Cell Mol Med. 2012 Sep 26;16(10):2301–10. doi: 10.1111/j.1582-4934.2012.01538.x (PMC3823423; doi:10.1111/j.1582-4934.2012.01538.x)

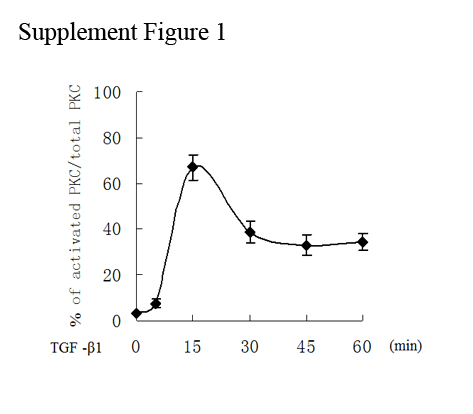

Supplement: Supplementary file 1 [file jcmm0016-2301-SD1.tif]

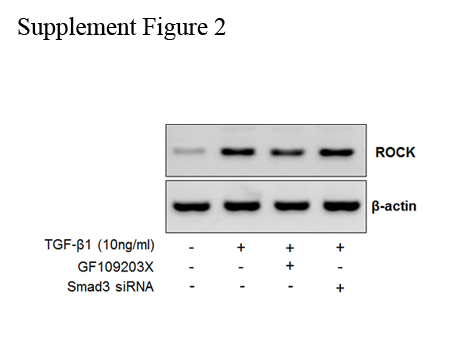

Supplement: Supplementary file 2 [file jcmm0016-2301-SD2.tif]

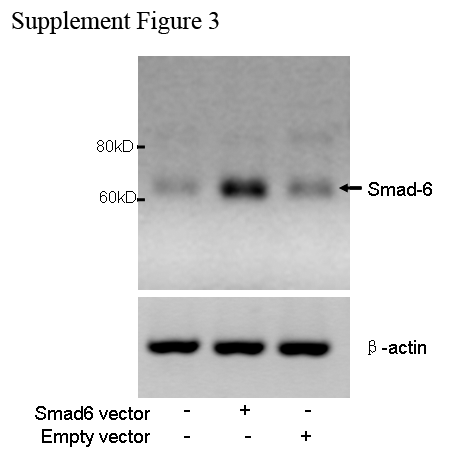

Supplement: Supplementary file 3 [file jcmm0016-2301-SD3.tif]

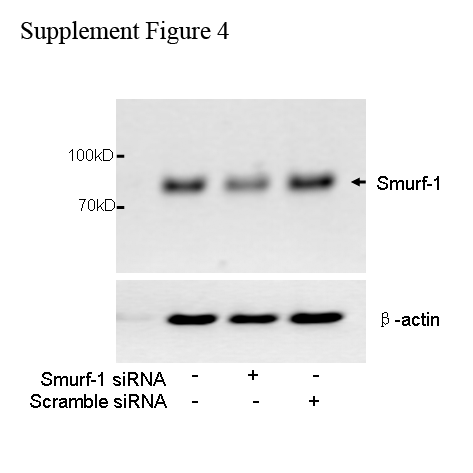

Supplement: Supplementary file 4 [file jcmm0016-2301-SD4.tif]

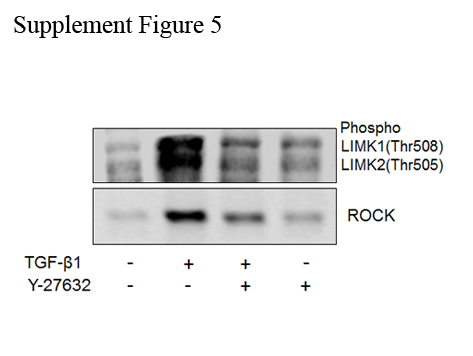

Supplement: Supplementary file 5 [file jcmm0016-2301-SD5.tif]
